# Learning from droplet flows in microfluidic channels using deep neural networks

**DOI:** 10.1038/s41598-019-44556-x

**Published:** 2019-05-31

**Authors:** Pooria Hadikhani, Navid Borhani, S. Mohammad H. Hashemi, Demetri Psaltis

**Affiliations:** 1 Optics Laboratory, School of Engineering, Swiss Federal Institute of Technology Lausanne (EPFL), CH-1015 Lausanne, Switzerland; 2 Computational Science & Engineering Laboratory, ETH Zurich, Zurich, Switzerland

**Keywords:** Mechanical engineering, Fluid dynamics

## Abstract

A non-intrusive method is presented for measuring different fluidic properties in a microfluidic chip by optically monitoring the flow of droplets. A neural network is used to extract the desired information from the images of the droplets. We demonstrate the method in two applications: measurement of the concentration of each component of a water/alcohol mixture, and measurement of the flow rate of the same mixture. A large number of droplet images are recorded and used to train deep neural networks (DNN) to predict the flow rate or the concentration. It is shown that this method can be used to quantify the concentrations of each component with a 0.5% accuracy and the flow rate with a resolution of 0.05 ml/h. The proposed method can in principle be used to measure other properties of the fluid such as surface tension and viscosity.

## Introduction

Machine learning is a framework that learns from the data without being programmed. Deep leaning is a specific approach of machine learning^1^ which has emerged in recent years as a powerful technique for a broad range of applications. Deep learning consists of several representation layers where each layer is obtained by the non-linear transformation of the previous layer^2^. Deep neural networks use the combination of these transformations to learn complex functions. In fluid mechanics, neural networks have been reported recently^3–5^ as tools that can help computational fluid mechanics simulations by mapping the estimates of low-resolution simulations to those with higher fidelity. In microfluidics, neural networks have been used to estimate various quantities for different applications^6–9^. Mahdi and Daoud^10^ used neural networks to predict the size of the droplets in an emulsion while Khor *et al*.^11^. used them to predict the stability of droplets in an emulsion. In our work, we employ neural networks to estimate fluid and flow parameters by observing the droplet formation process in a passive microfluidic chip. We extract this information by monitoring the flow of droplets with an optical microscope and training a neural network to obtain useful information from the recorded images. In one experiment, we trained a network to accurately measure the flow velocity at the inlet of the channel from droplet images. In a separate demonstration, a network was trained to identify the concentration of isopropanol in water in the droplet-forming solution. Our experiments demonstrate that DNNs can capture the complex nonlinear phenomena that result in the flow patterns and droplet shapes.

The flow rate is a parameter that affects droplet formation^12^ and can change the size, generation frequency, and pattern of droplets^13^. Similarly, the dilution ratio of isopropanol (IPA) in water affects the generation frequency and flow pattern of the droplets. Such effects can also be recognized by the DNN to measure the concentration. There are alternative methods for measuring the flow rate^14–17^ or the dilution ratio^18–21^ in a microfluidic chip. The two prominent methods for on-chip flow measurements are the Coriolis method^17^, based on mechanical oscillations that depend on the flow rate, and thermal measurement^16^, in which a heater and thermometer are used to measure temperature changes in the flow. Commercial Coriolis devices are available with 0.05 ml/h accuracy while thermal devices typically have an accuracy of 0.012 in the range of 0 to 0.12 ml/h. A thermal flow rate sensor with accuracy as low as 0.0001 ml/h has been reported but for a very small range (0 to 0.09 ml/h). All these alternative methods require integration of additional complex devices on the microfluidic chip whereas the method we describe requires only an optically transparent window and an external camera. In addition, measuring the flow rate of two-phase flows is a challenging task. A correction technique is needed to be applied to the measured values of the flow rate sensor in order to have an accurate measurement^22–24^. DNNs can be used to measure the flow rate of the desired phase in a two-phase flow. For the measurement of concentration, the Coriolis device can also be used^17^ with up to 0.25% accuracy. Optical methods (refractometry)^21^ have been demonstrated to have excellent accuracy (as low as 0.01% concentration of IPA versus 0.5% for our demonstration); however, fine resolution optical refractometers generally rely on resonant mechanisms which are difficult to integrate into microfluidic chips. Optical probes and DNNs can be implemented to measure compounds concentrations for applications such as ammonium concentration measurement in oceanography^25^, hydrocarbon concentration measurement in reacting flows^26^, concentration measurement in microfluidic mixers^27,28^, and measurement of contaminant concentrations in water^29^. From a practical point of view, an interesting feature of the method presented in this paper is that a single mechanism can in principle be used for the measurement of a variety of different quantities, such as surface tension or viscosity, in addition to the ones demonstrated here. The challenge is to design the system and the training process such that the effects due to an individual parameter of the system dominate the pattern of the droplets.

## Results

**Figure 1:**
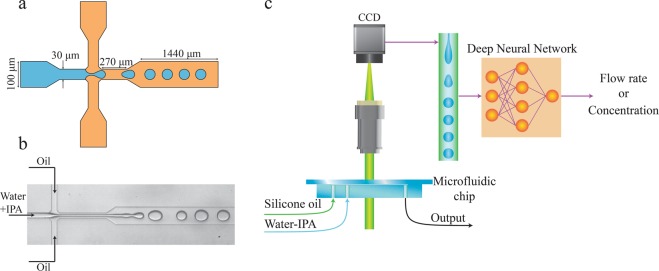
Using neural networks to measure fluidic properties from droplet flow patterns: (**a**) Schematic of the microfluidic channels (depth is 30 *μm*). (**b**) Image of droplet generation. Oil flows from the side channels and the water-IPA flows from the middle channel. (**c**) The two-phase pattern of the droplet generation in a microfluidic device contains information about the fluid and the flow properties. Neural networks are used to extract these properties while the main process stays intact.

**Figure 2:**
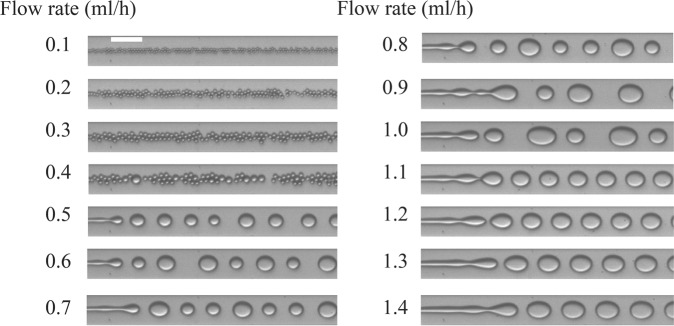
Images of droplet generation at different flow rates of the dispersed phase. The dispersed phase is water-IPA at a concentration of 5.5% and the continuous phase is silicone oil. The flow rate of the droplets are shown in the image and the silicone oil flow rate is 2 ml/h. Scale bar is 100 *μm*.

**Figure 3:**
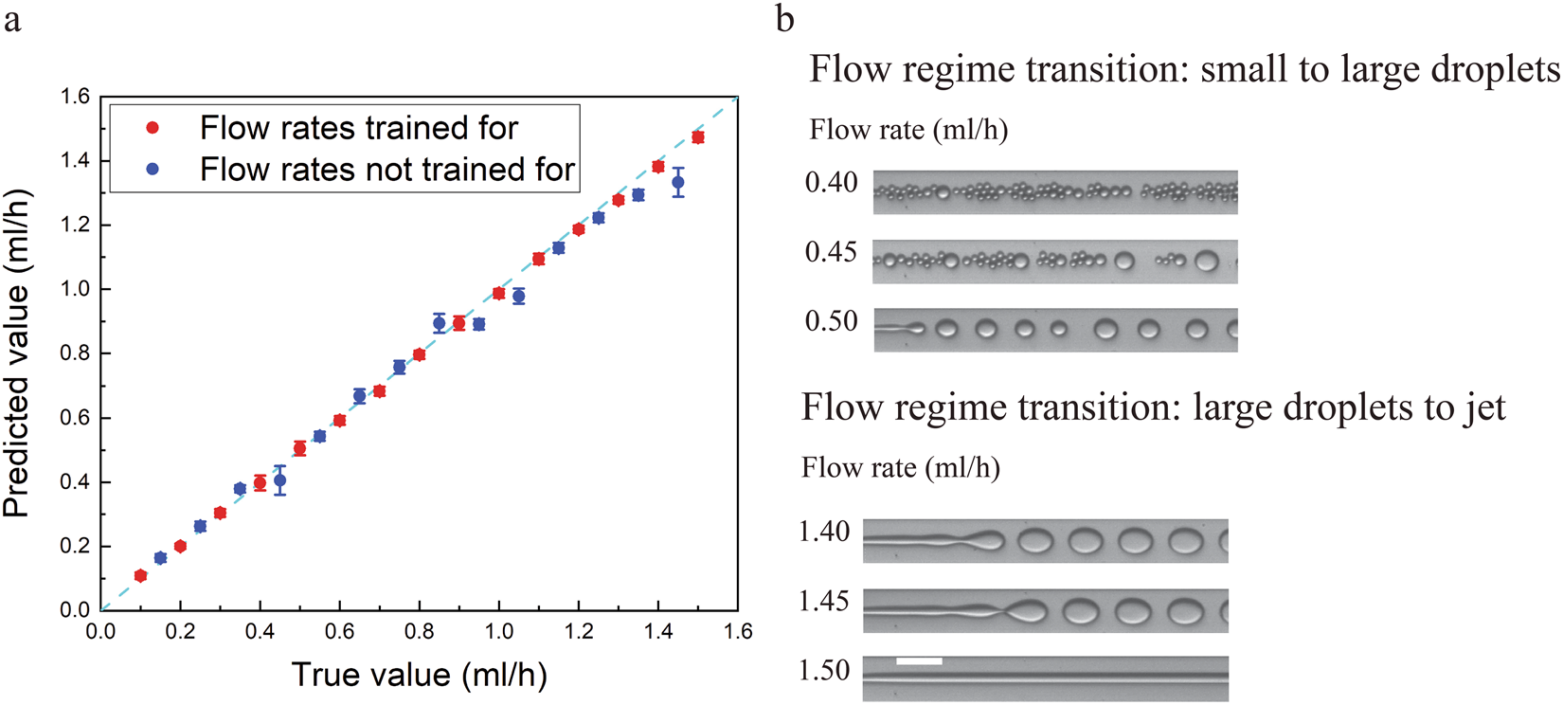
(**a**) Flow rate measurement: the measured results are compared with the true values for a water-IPA concentration of 5.5%. The red points are the flow rates for which the network is trained and the blue points are the new flow rates. Error bars show the standard deviation of the predicted values. (**b**) Examples of the flow regime transitions that occur at 0.45 ml/h and 1.45 ml/h flow rate of water-IPA. Silicone oil flow rate is 2 ml/h. The scale bar is 100 *μm*.

Figure 1 depicts the microfluidic device with input flows of silicone oil and water-IPA solutions. Droplets of the water-IPA solution are generated at the cross junction. Images of the droplets are captured using a wide-field microscope. Figure 2 shows examples of such images for an IPA in water concentration of 5.5% for different flow rates. A network with the structure shown in Fig. S2b is trained with 6000 images of the droplets (400 images for each flow rate) at flow rates ranging from 0.1 ml/h to 1.5 ml/h with 0.1 ml/h steps. The silicone oil flow rate is kept constant at 2 ml/h. The output of the network is a single unit whose analog value is trained to be the flow rate of the corresponding image. The trained network is tested with 2900 new images (100 images for each flow rate) recorded at flow rates ranging from 0.1 ml/h to 1.5 ml/h with 0.05 ml/h increments (Fig. 2). Therefore, the ability of the network in determining new flow rates is evaluated in addition to its ability to recognize flow rates from the training set. Figure 3a shows the results of the DNN for all concentrations. The mean error of the prediction is calculated by averaging the absolute relative difference between the predicted value and the true value. The mean errors for the trained and new flow rates are 2.9% and 5.7%, respectively. For non-trained flow rates, there are some cases in which the predicted flow rate is significantly different from the true value. This happens because a flow regime transition takes place as the flow rate is changed. Two examples of such transitions are shown in Fig. 3b. For example, the flow rate at 1.45 ml/h is not predicted correctly by the neural network because the flow pattern switches from individual droplets at a flow rate below 1.45 ml/h into a jet regime above this flow rate. The results of droplet flow rate measurement at a different silicone oil flow rate (1.5 ml/h) are shown in Fig. S4 which confirms the ability of the DNN in the flow rate measurement. We can improve the performance of the neural network by acquiring multiple recordings under different conditions that undergo the flow regime transitions at different flow rates. For example, we could include multiple channels on a single chip with different dimensions. We discuss this idea further in the following discussion on concentration measurement.

**Figure 4:**
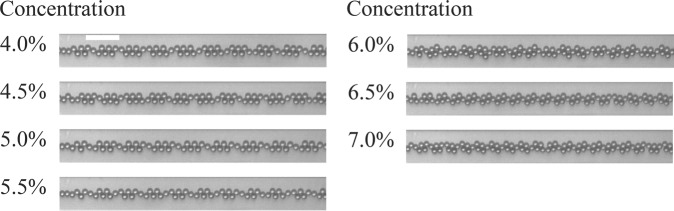
Images of droplet generation at different concentrations of the water-IPA solution where the concentration of the water-IPA solution is changing from 4.0% to 7.0% with 0.5% increment. The water-IPA and the oil flow rates are 0.2 ml/h and 1.0 ml/h, respectively. The scale bar is 100 *μm*.

**Figure 5:**
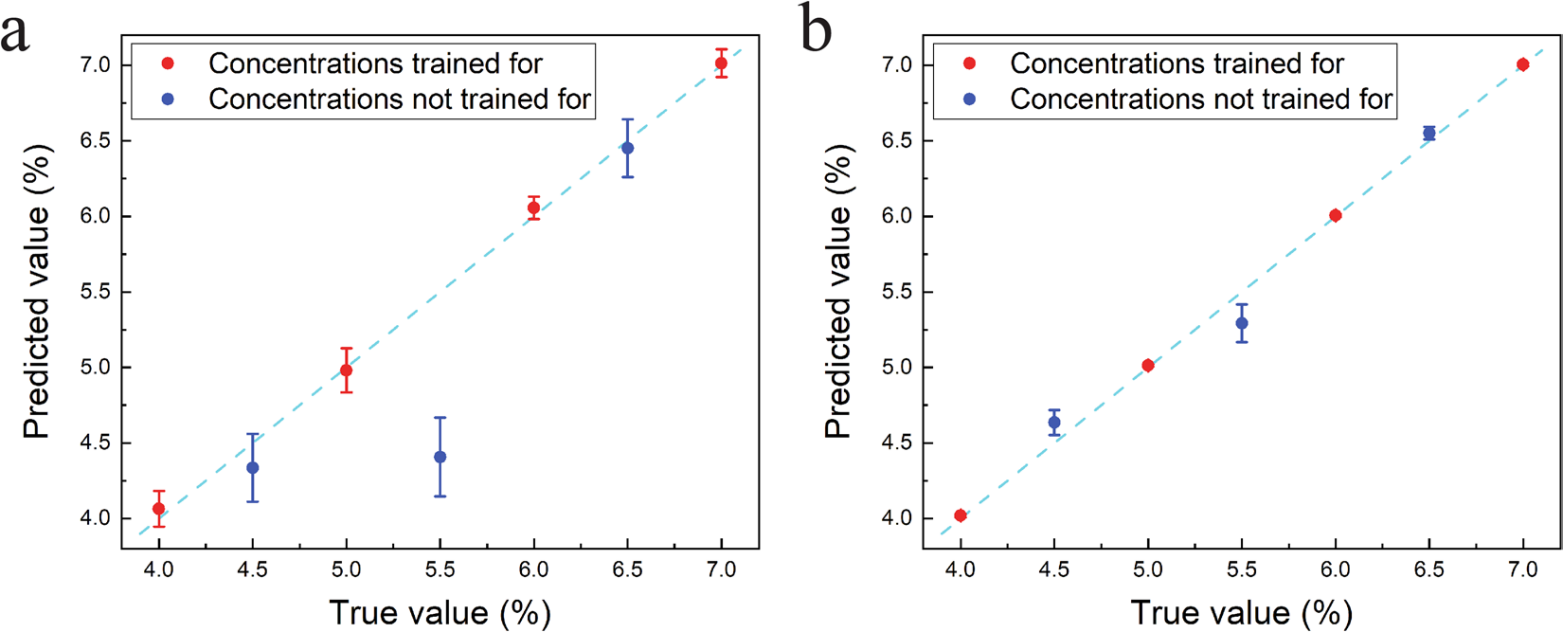
Predicted concentration by the DNN is compared to the true values. (**a**) Concentration prediction results using one DNN, and (**b**) concentration prediction results using six DNNs. The red points are the concentrations that the network is trained with and the blue points are the new concentrations. Error bars show the standard deviation for each case.

Figure 4 shows droplet flows at different concentrations of the water-IPA solution where the water-IPA flow rate and the oil flow rate are fixed at 0.2 ml/h and 1.0 ml/h, respectively. The DNN shown in Fig. S2b is trained with 3600 images of each of the four concentrations ranging from 4% to 7% with 1% increments. The trained network is tested with 400 images of each of the seven concentrations ranging from 4% to 7% with 0.5% resolution to evaluate the ability of the network in predicting new concentrations (4.5%, 5.5%, and 6.5%). The diagram of Fig. 5a compares the predicted values with the ground truth. The mean errors of the network for the trained and non-trained concentrations are 1.5% and 9.3%, respectively.

As we can see in Fig. 5a, the network correctly estimates the 4.5% and 6.5% concentrations but it cannot correctly identify the 5.5% concentration. This shows that the change in the pattern from 5.0% to 6.0% is not monotonic. This non-monotonic behavior is the result of the transition between different regimes of the droplet flow pattern due to the change in the concentration of IPA in the solution. This flow regime transition is described in more detail in the Supplementary Material Section (Fig. S3).

Since the flow regime transition is due to a small change in concentration, the DNN cannot predict accurate values close to this point. However, the concentration at which the transition occurs can be relocated by changing the flow rate of the fluid. In this way the outlier points originating from these transitions can be corrected. We tested this idea by recording images of the flow patterns for six different flow rates and using them to train six DNNs independently. We tested the system by feeding six images of the droplet flows from the six flow rate sets to the corresponding DNNs to generate six concentration estimates. Maximum three outliers are removed to reduce the standard deviation of the predicted values. The closest value to the average of the remaining values is taken as the predicted value for the concentration. Figure S5 shows the result of this procedure. The performance of the system can be enhanced further by recording multiple instances of the droplet patterns for each flow rate. We estimate the concentration by averaging the estimates obtained from the individual samples. This procedure reduces the variance of the estimates but it does not change the mean value. Using ten samples as an input, the mean error for concentrations that were included in the training set is reduced to 0.259% and for the concentrations outside the training set to 2.583%. Figure 5b compares the predicted values with true values.

As a final evaluation, we tested the sensitivity of the DNN results with respect to the microfluidic chip. The experiment for the concentration measurement was repeated for a new microfluidic chip but with the same design. Unfortunately, the DNNs that were trained to estimate the IPA concentration using data from the first chip did not give accurate readings for the new chip. When new DNNs were trained with data from the new chip only, the performance obtained was practically the same as for the first chip (Fig. S6 in the Supplementary Materials Section) even though the flow regime transitions occurred at different concentration levels. This indicates that the polydimethylsiloxane (PDMS) fabrication process of the microfluidic channels does not replicate the devices with sufficient precision for the DNNs to be used across different chips. We believe this is mainly due to variations in the PDMS wall properties after O_2_ plasma treatment and possibly small dimensional variations. A possible solution is to train the DNNs using data from multiple PDMS devices and make the measurements insensitive to the change of chips.

## Discussion

In this paper, we discuss how to measure fluidic properties using microfluidics and DNNs. We show that the classification of fluid properties using traditional image processing methods of the fluid flow patterns does not lead to satisfactory results (section S7 in Supplementary Materials Section). Using DNNs, the accuracy of the classification is drastically increased. Although image processing can be used to measure the droplets size, eccentricity, and velocity^30–32^, it is challenging to measure fluid properties such as concentration where the function that relates the effective parameters to the fluid properties is not known. DNNs are shown to be capable of learning the effective factors in two-phase droplet flow for the classification, thus highlighting the potential of deep learning in the microfluidics domain. We use a network with a single analog value output which corresponds to the value of a fluid property. We show that many fluidic properties and parameters can be inferred “on the fly” from this type of DNN, which receives its inputs from a simple imaging system. High accuracy prediction of the composition of the dispersed phase and flow rates are presented as two examples in our work.

The method described in this paper can be used to measure the flow rate even when the flow rate is changing within the device. This change in flow rate happens for instance in micro-reactors where gaseous products are generated or consumed in fluidic channels^33–36^. In these devices, it is important to measure the flow rate of the gas phase in order to determine their throughput and efficiency. Using DNNs is a suitable approach to measure the gas flow rate online. Moreover, DNNs measure the flow rate in the cases where the exact flow rate inside the microfluidic chip is unknown^37,38^. Furthermore, this method can be used to measure other fluid parameters. For instance, droplets in microfluidic channels are being used as the reaction media^39,40^. This application of droplet microfluidics requires the assessment of the droplet contents^41–43^. DNNs can be utilized for this purpose as chemical reactions change the properties of the droplets. Surface tension and viscosity measurements are other parameters that can be measured using DNNs. Many studies have investigated the effect of surface tension and viscosity on the droplet flow pattern^44–46^. In another approach, the changes in the patterns can be used in neural networks to obtain these influential properties.

One of the limitations of our method is the sensitivity of the result to channel dimensions, pressure, and temperature fluctuations. These parameters should be the same during the measurement and during training to achieve highly accurate results. Intelligent control of fluid parameters using machine learning^47^ is a recently developed approach that improves the repeatability of an experiment in a microfluidic device. By combining our approach with the control method of^47^, we can potentially maintain the same conditions of the training experiment and perform the measurement. In addition to this approach, we can train a DNN with inputs from a range of channel dimensions, temperatures, and pressures. A network trained with these inputs will predict more accurately because it has learned a range of parameter values. However, if the range of fluctuations increases, more complex design of the microfluidic device is needed to provide more information from the fluid to the network during the training.

This network, in conjunction with a microfluidic chip, can be potentially used for the measurement of multiple fluid/flow properties at the same time. However, it should be taken into consideration that it is possible to reach the same result by changing two parameters separately. In this case, the information in the pictures is not enough to distinguish these parameters. The voting system used for the concentration measurement is an approach to prevent incorrect measurement under these circumstances. This system considers outputs from multiple networks and determines the correct concentration of the liquid by neglecting outlier output(s). In another approach, the addition of obstacles in the microfluidic channel and changing the geometries of the channels can force droplets to undergo different deformations, which would increase the information present in the images and especially be useful for surface tension and viscosity measurement. Fast and reliable readout of such parameters is important in many microfluidic applications such as chemical synthesis in multiphase flow systems^48,49^, cell culture^50^, and electrochemical microreactors^33,35^.

We remark that although many fluidic properties can be measured using traditional sensors physically connected to the chip, their integration adds to the cost, size, and complexity of a microfluidic-based system. The presence of such cumbersome peripheral hardware is in fact what makes most of today’s microfluidic devices best described as “Chip-in-a-lab” instead of a “Lab-on-a-chip”^51,52^. We believe that many functionalities of these physical systems can be integrated into a compact on-chip imaging system^53,54^ that benefits from a pretrained learning algorithm to decode the rich information encoded in the images.

## Methods

A flow-focusing geometry is used to create droplets of water-IPA (Fisher Scientific UK) solution in silicone oil (viscosity 10 cSt, SIGMA-ALDRICH). The water-IPA mixture enters the main channel from the middle and the oil flows from the sides, as illustrated in Fig. 1. The microfluidic chip containing this geometry is fabricated from PDMS using standard photolithography. The PDMS is plasma bonded to a glass slide to seal the microfluidic channels and subsequently heated at 80 °C for 48 hours to retrieve its hydrophobic properties.

For the concentration measurements, the IPA volume concentration in water is varied in 0.5% steps from 4% to 7%. For the flow rate measurements, the dispersed phase is a solution of 5.5% IPA in water. The flow rate of the water-IPA solutions is varied in 0.05 ml/h steps from 0.1 ml/h to 1.5 ml/h. Considering the precision of the pipettes and the syringes used, the accuracy of the IPA volume concentration in water is 0.08%. During the experiments, the inlet and the outlet temperatures were monitored and the mean temperature was 22.5 °C. The temperature variations were less than 1 °C during data acquisition, a negligible fluctuation which did not have a noticeable effect on the performance of DNNs.

There is one inlet for each liquid and one output for the two phases. The flow rates of the liquids are regulated using syringe pumps (Cronus Sigma 1000 Series). The images are captured by a high-speed camera (Photron FASTCAM Mini UX100) and the image resolution is 1.8 μm/pixel. Images are recorded from the channel in a region downstream of the flow focusing geometry in which the channel dimensions are 100 μm by 1440 μm and 30 μm depth. The smallest droplet in experiments is 12 μm in diameter and is therefore fully resolved in the images. For each experiment, 4000 images are recorded at 50 frames per second. An image denoising algorithm from the OpenCV library^55^ and an edge detection algorithm from Python’s scikit-image library (the standard deviation of the Gaussian filter is set to 1.2) are applied to the images to detect the droplet boundaries and remove the effect of the background and the light profile in the images. Image processing used in this paper is explained in more detail in the Supplementary Materials Section. This procedure is important to make sure that the DNN does not learn something related to the geometry of the channel or the light intensity. The processed images are used to train the DNNs. The structure of the DNNs is shown in the Supplementary Materials Section (Fig. S2).

## Supplementary information

Supplementary Information

## Data Availability

The data and the code used for this study are available from the corresponding author on reasonable request.
