# Supplementary material for: Learning from droplet flows in microfluidic channels using deep neural networks
Source: Sci Rep. 2019 May 31;9:8114. doi: 10.1038/s41598-019-44556-x (PMC6544611; doi:10.1038/s41598-019-44556-x)
Supplement: Supplementary file 1 — Supplementary Information [file 41598_2019_44556_MOESM1_ESM.docx]

**Supplementary Information**

**Learning from droplet flows in microfluidic channels using deep neural networks**

Pooria Hadikhani^1*^, Navid Borhani^1^, S. Mohammad H. Hashemi^1,2^, Demetri Psaltis^1^

^1^ Optics Laboratory, School of Engineering, Swiss Federal Institute of Technology Lausanne (EPFL), CH-1015, Lausanne, Switzerland.

^2^ Computational Science & Engineering Laboratory, ETH Zurich, Zurich, Switzerland.

email: [pooria.hadikhani@epfl.ch](mailto:pooria.hadikhani@epfl.ch)

**S1. Image preparation for the neural network**

OpenCV and Python’s scikit-image libraries are used in python to prepare the input images of the neural network. The image from the experiment is cropped to achieve an image without the channel walls (Figure S1.1). From OpenCV, fastNlMeansDnoising is applied to reduce noises in the image as shown in Figure S1.2. In this procedure, the template window size and search window size are set to 7 and 21, respectively. Finally, the Canny edge detector filter is used to detect droplets boundaries as shown in Figure S1.3. The standard deviation of the Gaussian filter used in the Canny edge detector is set to 1.2.

| 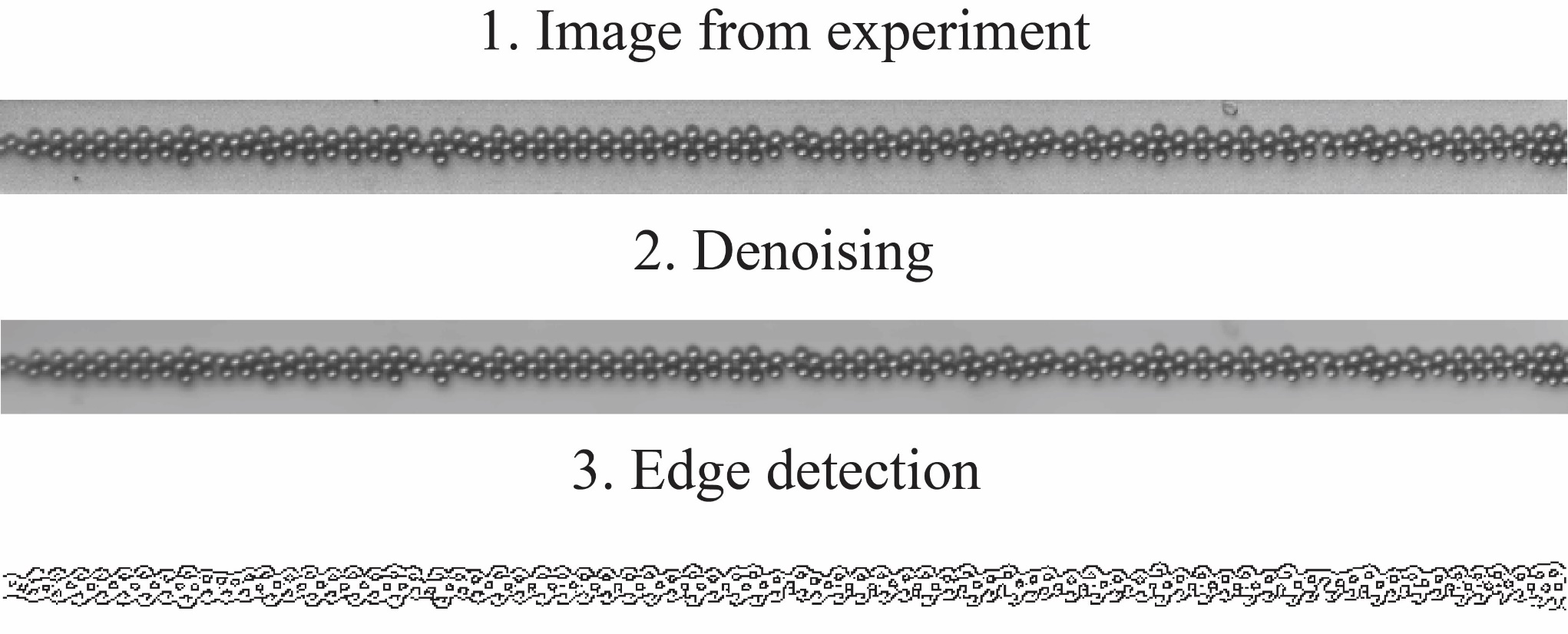 |
| --- |

Figure S1. Image processing procedure: 1. cropped image from the experiment, 2. Applying denoising algorithm to remove the noise in the image, 3. Result of edge detection algorithm in detecting droplets boundaries.

**S2. Neural Network Structure**

The Tensorflow library is used to implement the neural networks [1]. For the classification of the images based on the concentration, a convolutional neural network (CNN) with two convolutional layers followed by two fully connected layers is used [2]. The rectified linear activation function is applied to all layers. Each convolutional layer is accompanied by a max pooling layer that reduces the spatial size by half. The structure of this network is depicted in Figure S2a. A dropout layer (40% rate) is added after the fully connected layer to prevent the overfitting by setting the randomly chosen neurons to zero [3]. The learning rate and the batch size are set to 0.001 and 40. A gradient descent optimizer is applied to minimize the cross-entropy cost function. 90% of the images are used for the training and the validation of the network and 10% of them are used for the testing. The accuracy of this network is the proportion that classified correctly.

Another CNN, shown in Figure S2b, is used to predict the concentration of the solutions that the network has not seen. This CNN contains three convolutional and two fully connected layers, where the rectified linear activation function is applied to the output of each layer. There are three max-pooling layers for three convolutional layers and there is a dropout layer (50% rate) after the fully connected layer to reduce the overfitting. The output of this network has one neuron that gives the concentration value. An Adam optimizer is used to minimize the mean squared error cost function. Initially, the learning rate is 0.001 and decreased by half every 50 epochs.

| 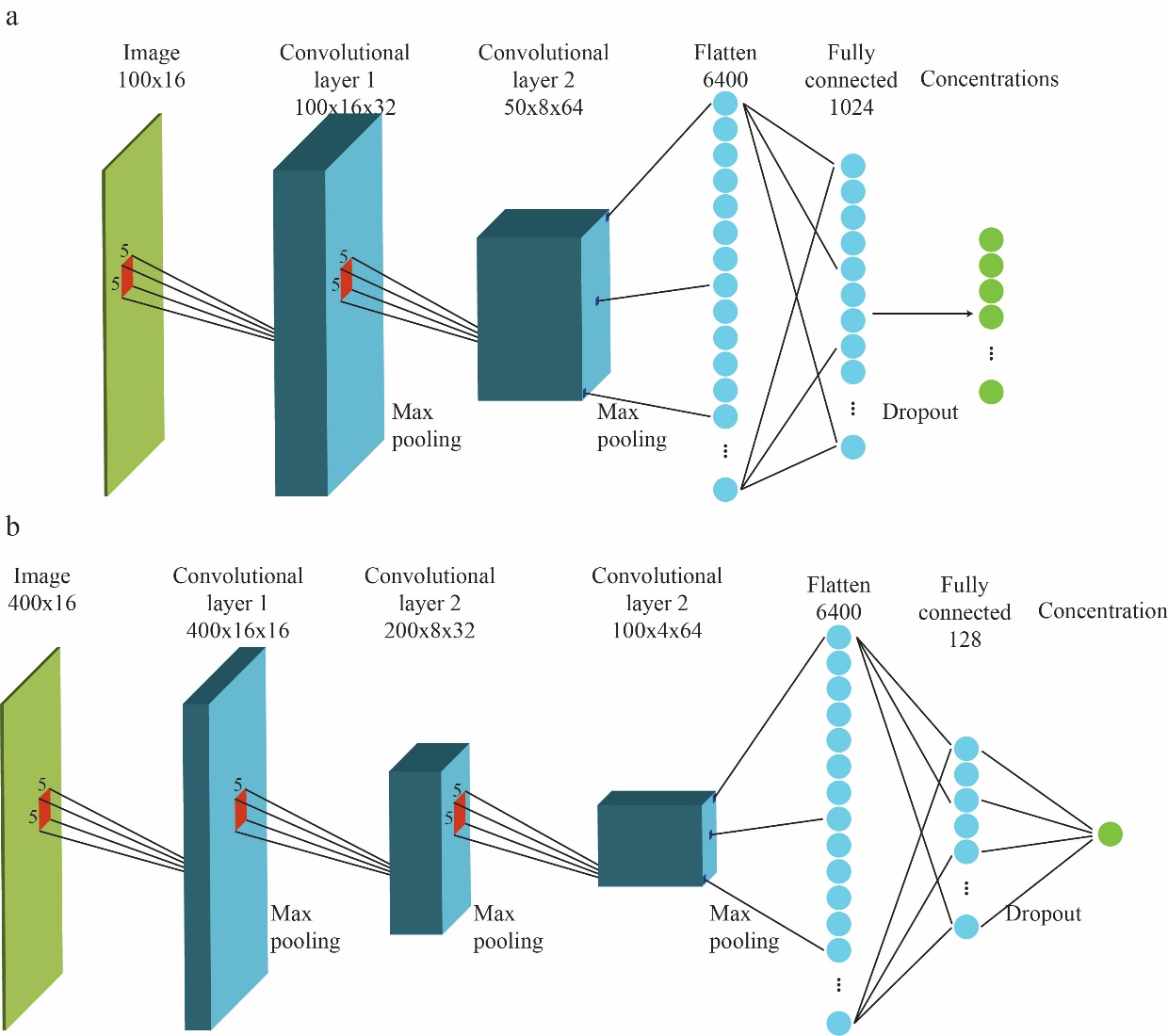 |
| --- |

Figure S2. The structure of the convolutional neural networks: (a) A classifier network with two convolutional layers and two fully connected layers is used to classify the images based on their concentrations. The convolutional filters are $5\times5$ and there is a max pooling layer after each convolutional layer. A dropout layer is used before the last layer. (b) A regression neural network is implemented to predict the concentration of the inputs. This network consists of three convolutional layers followed by a fully connected layer. There is a max pooling layer after each convolutional layer and a dropout layer after the fully connected layer.

**S3. Flow pattern**

Figure S3 shows the two-phase flow pattern at two different flow rates and for two different concentrations. As we can see in this figure, droplet generation patterns are changing by the flow rate or the concentration. The five symbols in Figure S3 correspond to droplet  flow patterns that represent the five flow regimes that were observed in this case. The pictures in Figure S3c are examples for  each of the phases.

| 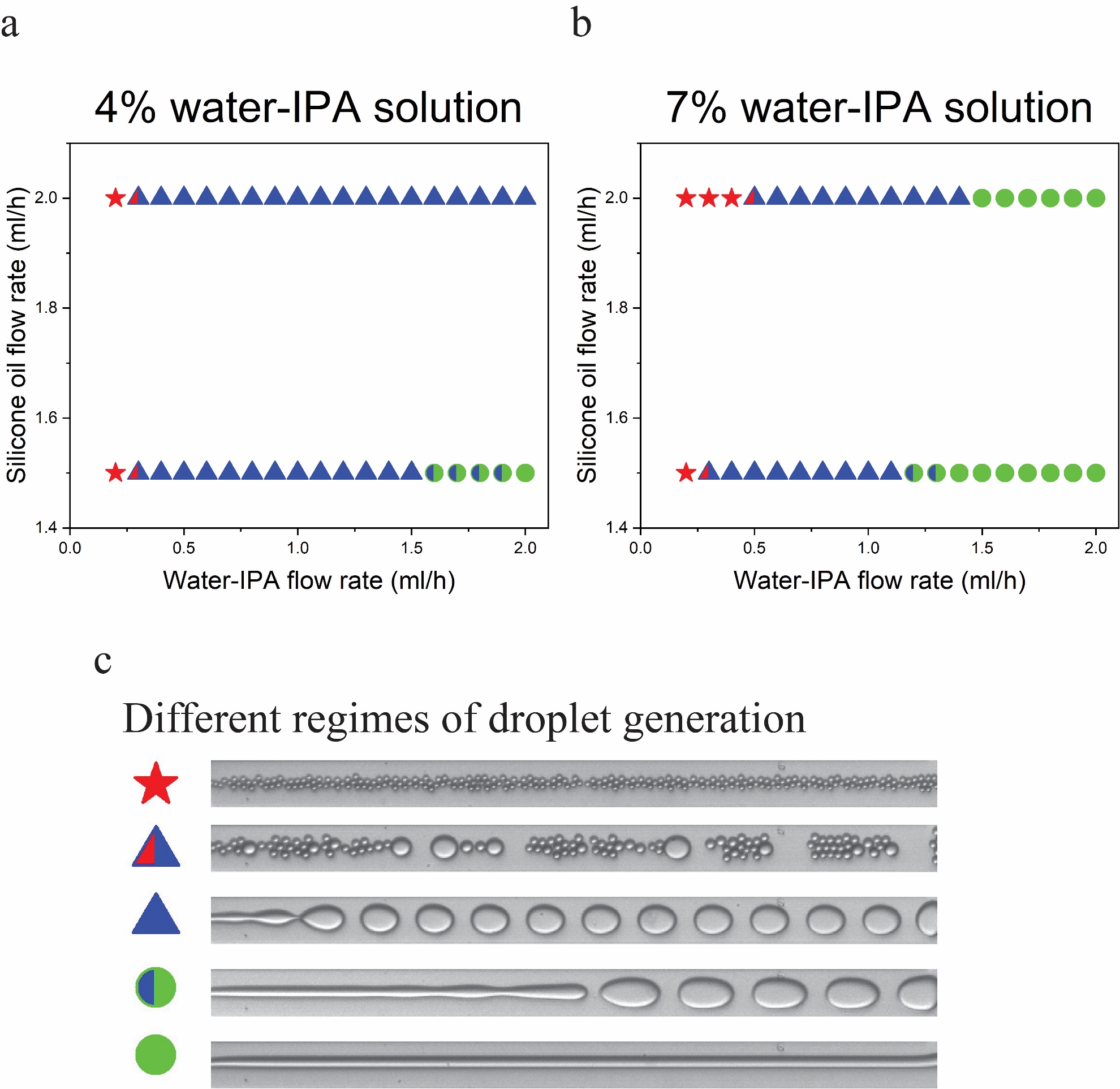 |
| --- |

Figure S3. Flow pattern of droplet flows: (a) 4.0% water-IPA concentration. (b) 7.0% water-IPA concentration. (c) for two different concentrations: The phase transition happens by changing the flow rate or the concentration.

**S4. Flow rate measurement at silicone oil flow rate of 1.5 ml/h**

| 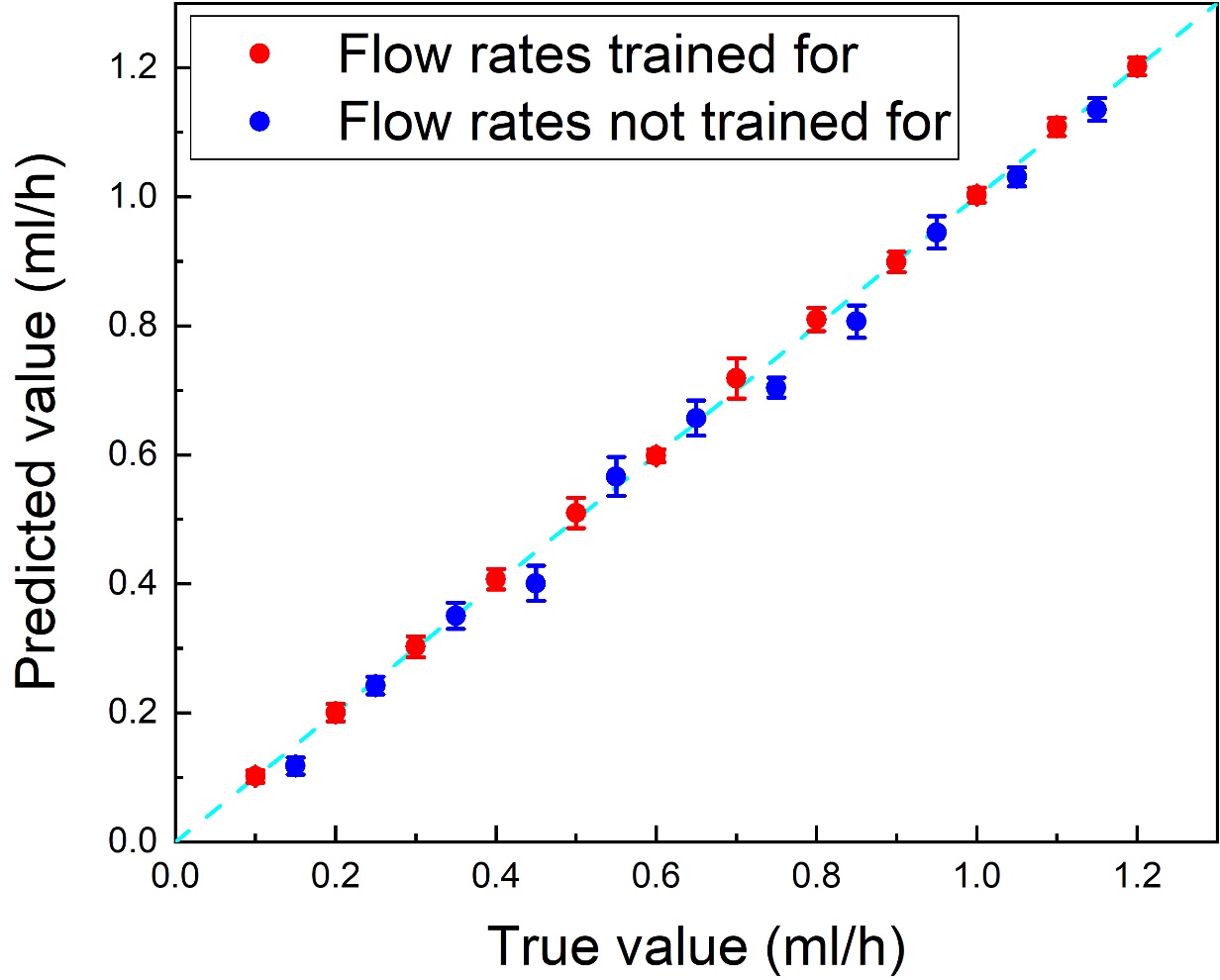 |
| --- |

Figure S4. Water-IPA flow rate measurement at silicone oil flow rate of 1.5ml/h.

**S5. Concentration prediction using six networks and one image input**

| **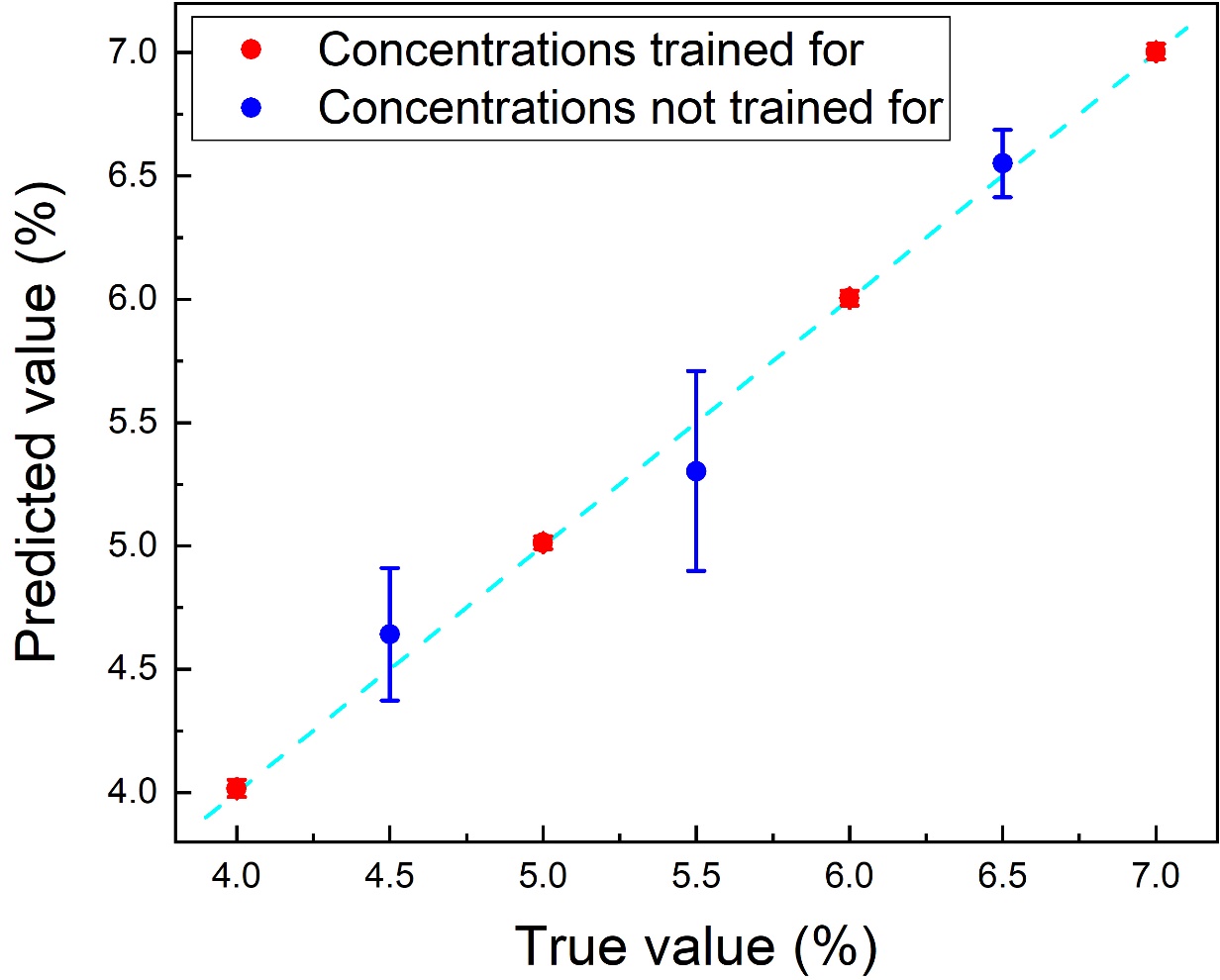** |
| --- |

Figure S5. Concentration prediction results using six DNNs and one image input. The red points are the concentrations that the network is trained with and the blue points are the new concentrations. Bars show the standard deviation for each case.

**S6. Results of the repeated experiment for the concentration measurement**

| 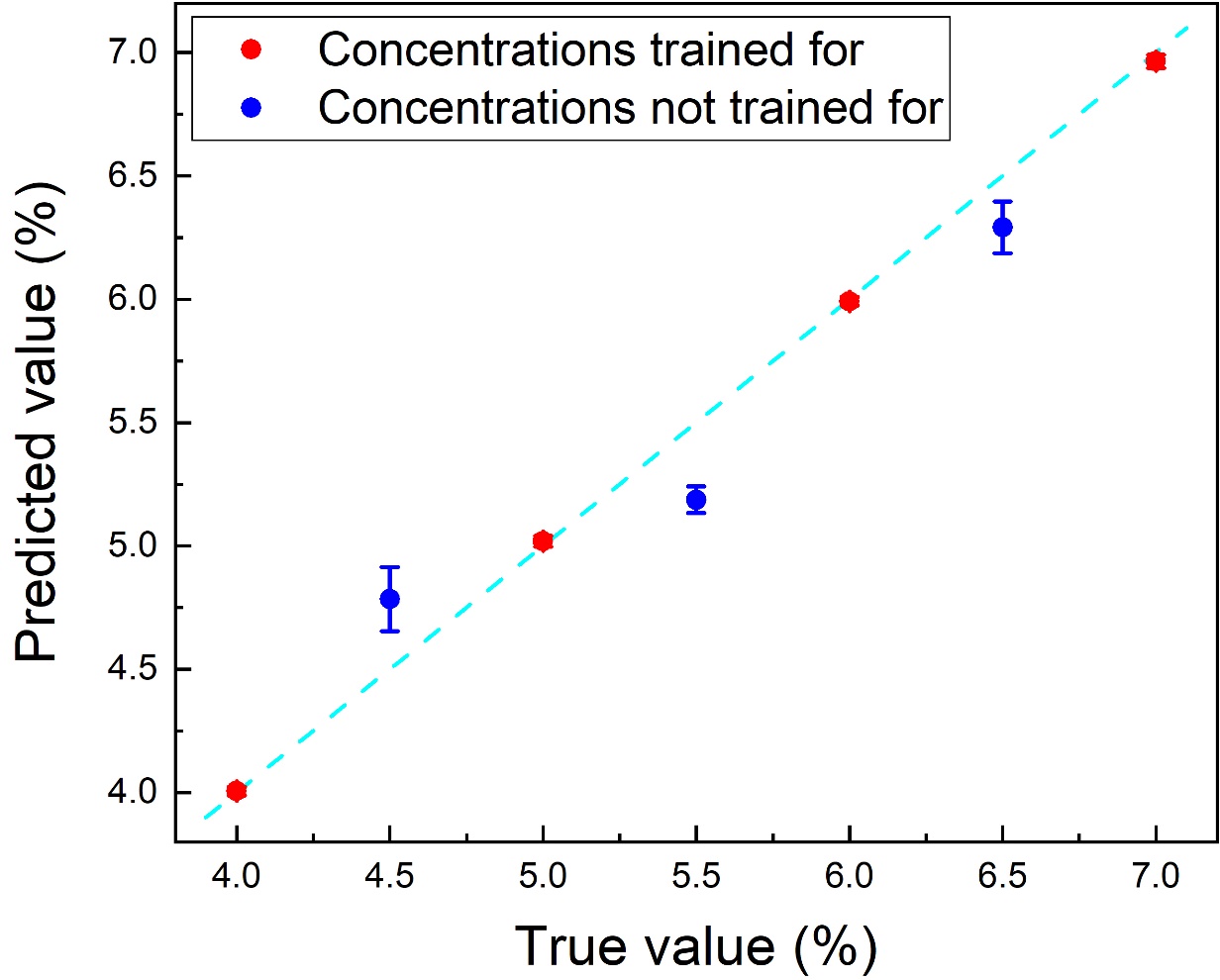  Figure S6. The diagram shows the results of applying the proposed method to the repeated experiment for the concentration measurement. |
| --- |

**S7. Comparison between a clustering algorithm (nearest neighbor) classification and neural network classification**

In this section, we extract two features of the droplet generation regimes and use them for determining the water-IPA concentration ranging from 4.0% to 7.0%. The extracted features are the number of the generated droplets (area is 100$\mu m$ by 1440$\mu m$) in one snapshot and their radius. A simple clustering method is applied to these features to classify each image. The circle Hough Transform (CHT) is applied to each image to detect the circles. Based on the outcome, each image is labeled by the number of the droplets and the mean value of their radii.

A collection of points in radius-number space is presented in Figure S6 by extracting the data from 25200 images. The new image is classified in the class of the closest point clusters. 2800 new images are classified in this way and the obtained accuracy is 54%. The reason for such a low accuracy is the overlap between the data of the different classes. In addition, we remark that the classification is more challenging for droplet generation regimes where the droplets are not circular.

| 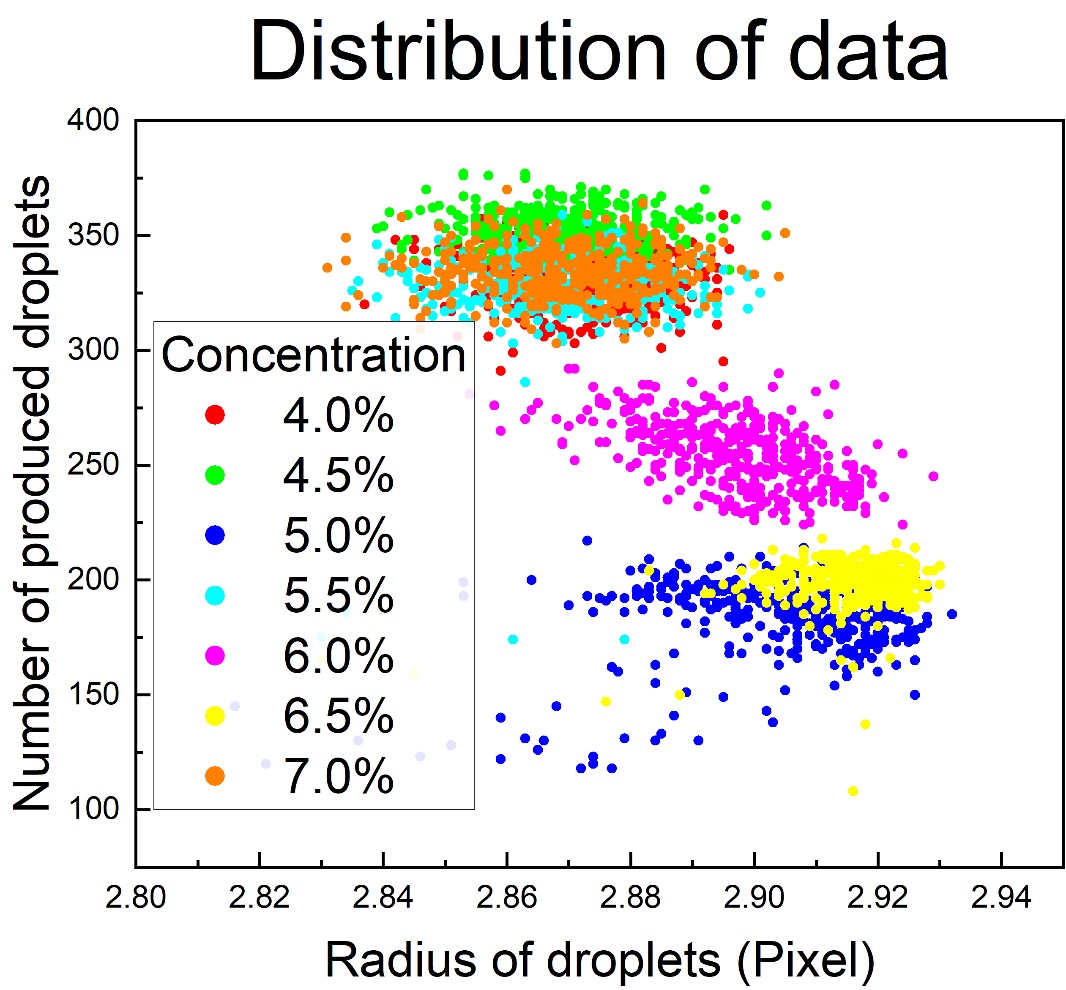 |
| --- |

Figure S7. Distribution of the dataset where the number and the radii of the droplets are presented for different concentrations.

Classification based on only the radius and the number of droplets is unreliable. More features must be extracted from the images for a better accuracy. DNN is an alternative approach for feature-based classification. Figure S7 shows some of the features that are detected by the convolutional layers of a DNN. In the first part of this figure, images of two different concentrations are shown in the experiment row. The second part shows the images after applying the edge detection algorithm to these experimental images. In the third part, each image shows the difference between the convolutional layer outputs of the two different concentrations shown in the first part. Figure S7a shows that the DNN detects the width of the droplets cluster as one of the features for the small droplets. The local number density of droplets is another feature that the DNN discovers as shown in Figure S7b. In this image, the brighter spots show that the number density of droplets in one image is higher than the other image in those positions. For the big droplets, two features detected by DNN are the droplets (Figure S7c), and the liquid jet (Figure S7d). Figure S7c shows that the DNN removes the liquid jet and only detects the boundaries of the droplets where features like periodicity, droplet size, and the droplet deformation can be extracted. On the other hand, in Figure S7d, the DNN highlights the liquid jet and extracts the length and the width of the jet as features.

| 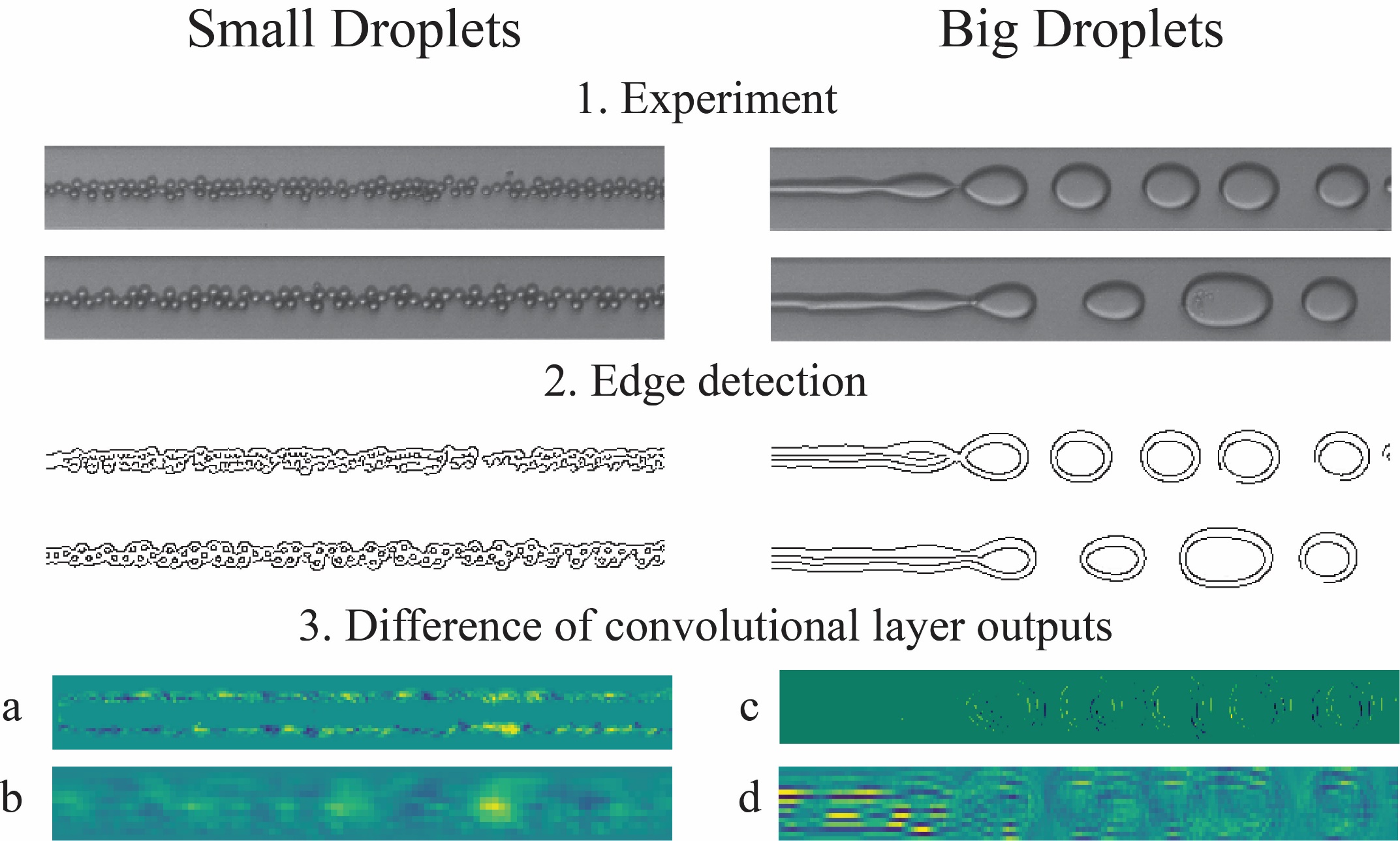 |
| --- |

Figure S8. Droplet images and the features detected by the DNN: 1. The images of the droplet generation from the experiment for small and big droplets at two different concentrations. 2. The results of the edge detection algorithm applied to the images from the experiment. 3. Difference of convolutional layer outputs for two different concentrations: (a) the DNN detects the width of the droplet clusters, (b) the local number density of the droplets are identified as the brighter regions indicate higher number of droplets in one image than the other, (c) the DNN extracts the leading edges of the droplets that represent the number of droplets and their periodicity, (d) the enhanced edges of the boundaries with an emphasis on the liquid jet which is detected by brighter color.

The DNN classifier [3] (illustrated in Figure S1a) is employed to classify the images of droplet generation at concentrations ranging from 4.0% to 7.0%. The DNN accuracies is 97.4%. The convergence and the loss diagrams of both cases are drawn in Figure S8. The DNN achieves a higher accuracy compared to the classification based on two features.

| 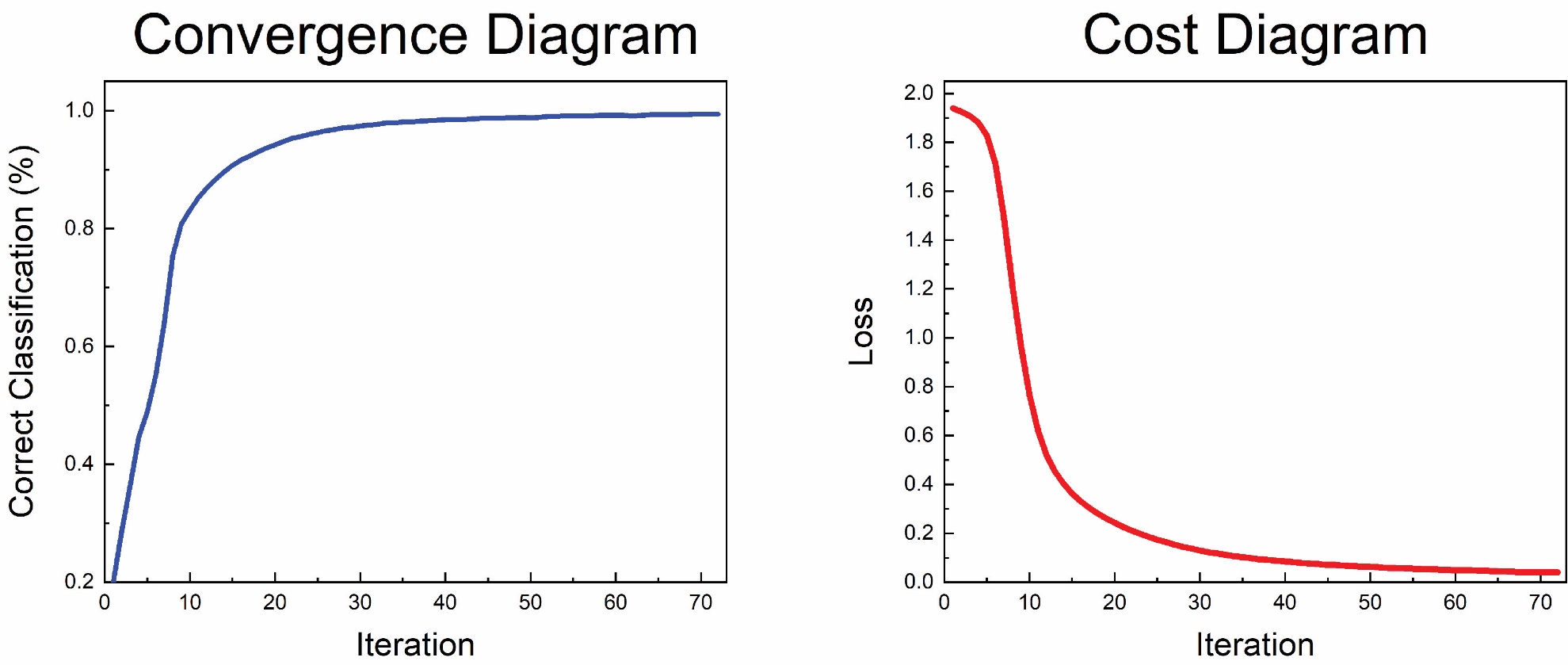 |
| --- |

Figure S9. The convergence plot and the cost diagram. The cost function is calculated using cross entropy loss.

**References**

1. Abadi, Martín, Paul Barham, Jianmin Chen, Zhifeng Chen, Andy Davis, Jeffrey Dean, Matthieu Devin, Sanjay Ghemawat, Geoffrey Irving, and Michael Isard. Tensorflow: a system for large-scale machine learning. in OSDI. 2016.

2. Krizhevsky, Alex, Ilya Sutskever, and Geoffrey E Hinton. Imagenet classification with deep convolutional neural networks. in Advances in neural information processing systems. 2012.

3. Simonyan, Karen and Andrew Zisserman, Very deep convolutional networks for large-scale image recognition. arXiv preprint arXiv:.04482, 2014.
